# Targeting cancer stem cell plasticity in triple-negative breast cancer

**DOI:** 10.37349/etat.2023.00190

**Published:** 2023-12-11

**Authors:** Zhengwang Guo, Shuyan Han

**Affiliations:** Institute of Experimental Endocrinology and Oncology “G. Salvatore”-National Research Council (IEOS-CNR), Italy; Key Laboratory of Carcinogenesis and Translational Research (Ministry of Education), Department of Integration of Chinese and Western Medicine, Peking University Cancer Hospital & Institute, Beijing 100142, China

**Keywords:** Triple-negative breast cancer, cancer stem cell, plasticity, therapeutic targets

## Abstract

Triple-negative breast cancer (TNBC) is a highly aggressive breast cancer subtype with limited treatment options. Cancer stem cells (CSCs) are thought to play a crucial role in TNBC progression and resistance to therapy. CSCs are a small subpopulation of cells within tumors that possess self-renewal and differentiation capabilities and are responsible for tumor initiation, maintenance, and metastasis. CSCs exhibit plasticity, allowing them to switch between states and adapt to changing microenvironments. Targeting CSC plasticity has emerged as a promising strategy for TNBC treatment. This review summarizes recent advances in understanding the molecular mechanisms underlying CSC plasticity in TNBC and discusses potential therapeutic approaches targeting CSC plasticity.

## Introduction

Breast cancer is a complex and heterogeneous disease that significantly threatens women’s health [1]. Triple-negative breast cancer (TNBC) is a subtype of breast cancer that is characterized by the lack of estrogen receptor (ER), progesterone receptor (PR), and human epidermal growth factor receptor 2 (HER2) expression [2]. It accounts for 10–15% of all breast cancer and has the worst prognosis. TNBC is a heterogeneous disease with diverse biological and clinical characteristics. It lacks targeted therapy options and has aggressive behavior, a higher recurrence rate, and a poorer prognosis than other breast cancer subtypes [3].

Cancer stem cells (CSCs) are a subpopulation of tumor cells with self-renewal and differentiation abilities [4], which play a crucial role in breast cancer initiation, maintenance, progression, metastasis, and treatment resistance [5]. CSC plasticity refers to the ability of CSCs to switch between CSC and non-CSC states in both directions, depending on the regulation of their self-renewal and differentiation, and it represents a significant therapeutic hurdle for cancer treatment [4, 6]. The plasticity of CSCs leads to changes in cell phenotype and function, enabling TNBC cells have higher tumorigenic and metastatic potential, to adapt and resist conventional anti-cancer therapies, thus resulting in tumor progression and recurrence. Therefore, targeting CSCs plasticity has emerged as a promising strategy for developing effective therapies to improve TNBC prognosis and overcoming treatment resistance. Researchers are searching for new therapeutic strategies targeting CSCs in TNBC.

The plasticity of CSCs is regulated by a complex network of signaling pathways, including Notch, Wnt/β-catenin, and Hedgehog, which are activated by interactions with the tumor microenvironment (TME) [7, 8]. In addition, epigenetic modifications, such as DNA methylation and histone modification, also affect CSC plasticity in TNBC [9, 10]. Despite the significant progress that has been made in understanding the mechanisms underlying CSC plasticity, there is still a critical need for new therapeutic strategies to target CSCs in TNBC [4]. This review will discuss the current knowledge regarding CSC plasticity in TNBC and its regulation in the TME. It will also highlight recent advances in developing targeted therapies for CSCs, including those targeting key signaling pathways and epigenetic modifiers [11, 12]. Finally, the challenges of translating preclinical research findings into clinical practice are discussed and future research directions in this area are suggested.

## CSCs in TNBC

CSCs have been identified in most solid tumors, including breast cancer, and are believed to play crucial roles in tumor initiation, development, relapse, and therapeutic resistance [13]. In TNBC, CSCs have been shown to have higher tumorigenicity and metastatic potential compared to non-CSCs [14, 15]. Moreover, CSCs are thought to be responsible for the resistance of TNBC to chemotherapy and radiation therapy [16].

Specific surface markers and intracellular proteins have been used for CSC identification in TNBC, such as CD24, CD44, and aldehyde dehydrogenase 1 (ALDH1) [14, 17]. For example, CD24^–/low^/CD44^+^ cells have been shown to have CSC properties in TNBC [18]. Meanwhile, CSCs can also be isolated by cell surface biomarkers such as extensively used epithelial cell adhesion molecule (EpCAM) and CD90 through cell sorting technology [19]. However, some CSC surface markers are shared with normal stem cells, and different studies have reported inconsistent results regarding the expression and prevalence of these markers in TNBC [20].

CSCs have also been implicated in developing TME [21]. CSCs can interact with stromal cells, such as cancer-associated fibroblasts (CAFs) and immune cells, to promote tumor growth, invasion, angiogenesis, and immune evasion [22]. Moreover, CSCs can secrete cytokines and growth factors that modulate TME, leading to immune suppression and tumor-promoting inflammation [23].

## Characteristics of CSCs plasticity

One of the hallmarks of CSCs is their plasticity, which refers to the ability of cells to switch between different states under various stimuli or microenvironmental cues, such as hypoxia, inflammation, and therapy-induced stress [24]. CSCs exhibit a high degree of plasticity, allowing them to adapt to changing environmental conditions and contribute to tumor heterogeneity and therapeutic resistance.

Plasticity has been implicated in CSC properties, including self-renewal, differentiation, and tumor-initiating ability. Studies have provided valuable insights into the plasticity-mediated regulation of CSCs in TNBC. For instance, one study found that TNBC CSCs exhibited high plasticity, allowing them to switch between non-CSC and CSC states in response to environmental stresses [18]. Another study demonstrated that the plasticity of TNBC CSCs was driven by epigenetic modifications, especially histone acetylation, and methylation [25].

CSCs plasticity plays a crucial role in tumor progression, metastasis, and treatment resistance in TNBC. The highly plastic behavior of CSCs affects the treatment and prognosis of TNBC through multiple ways, contributing to the heterogeneity of TNBC tumors, being associated with the acquisition of epithelial-mesenchymal transition (EMT) phenotype, undergoing phenotypic changes in response to therapy and allowing tumor cells to survive and regenerate, and others [26].

CSCs can undergo phenotypic and functional changes, leading to the acquisition of more aggressive and malignant behaviors. Cellular process and CSC formation can lead to tumor cell plasticity [27]. For example, in breast cancer, tumor cells can undergo EMT, a process by which epithelial cells acquire mesenchymal characteristics, transdifferentiates through the generation of CSCs following the EMT [28]. EMT has been shown to enhance tumor cell migration, invasion, and resistance to chemotherapy and radiation therapy [29]. In addition, tumor cells can also undergo a mesenchymal-epithelial transition (MET), a process by which mesenchymal cells revert to an epithelial phenotype. MET has been associated with the formation of metastatic lesions and increased CSC plasticity [30]. Furthermore, CSC plasticity can be modulated by various signaling pathways and transcription factors, such as Notch, Wnt/β-catenin, and SRY-box transcription factor 2 (SOX2) [31]. These pathways can regulate CSC self-renewal and differentiation and their interactions with the TME.

## Signaling pathways involved in CSCs plasticity in TNBC

Several signaling pathways have been implicated in regulating CSC self-renewal and differentiation. In TNBC, the plasticity of CSCs is regulated by various signaling pathways, including Notch, Wnt/β-catenin, Hedgehog, phosphatidylinositol 3-kinase (PI3K)/protein kinase B (Akt), and transforming growth factor-β (TGF-β), and others [32, 33]. These pathways can regulate the plasticity of CSCs and their interactions with the TME. Understanding the mechanisms underlying CSC functions in TNBC may lead to the development of novel CSC-targeted therapies for this breast cancer subtype.

Activation of the Notch pathway has been shown to promote CSC self-renewal and maintenance, and facilitate the transition between epithelial and mesenchymal states [34]. The Wnt/β-catenin pathway has also been implicated in regulating CSC plasticity by promoting self-renewal and differentiation [35]. In addition, activation of the Hedgehog pathway has been reported to promote CSC expansion and metastasis in TNBC [36]. The PI3K/Akt pathway regulates CSC survival and drug resistance in TNBC [37]. Furthermore, the TGF-β pathway is critical in promoting EMT and enhancing CSC pluripotency [38].

Targeting these signaling pathways has emerged as a promising approach for reducing plasticity and improving therapeutic outcomes in TNBC. For instance, inhibition of the Notch pathway has been shown to suppress TNBC growth and decrease the CSC population [39]. Similarly, targeting the Wnt/β-catenin pathway can inhibit CSC self-renewal and metastasis in TNBC [40]. Inhibition of the Hedgehog pathway reduces CSC frequency and sensitivity to chemotherapy [41]. Moreover, targeting the TGF-β pathway has been shown to sensitize TNBC cells to chemotherapy and inhibit the EMT process [42].

## TME and CSC plasticity in TNBC

**Figure 1:**
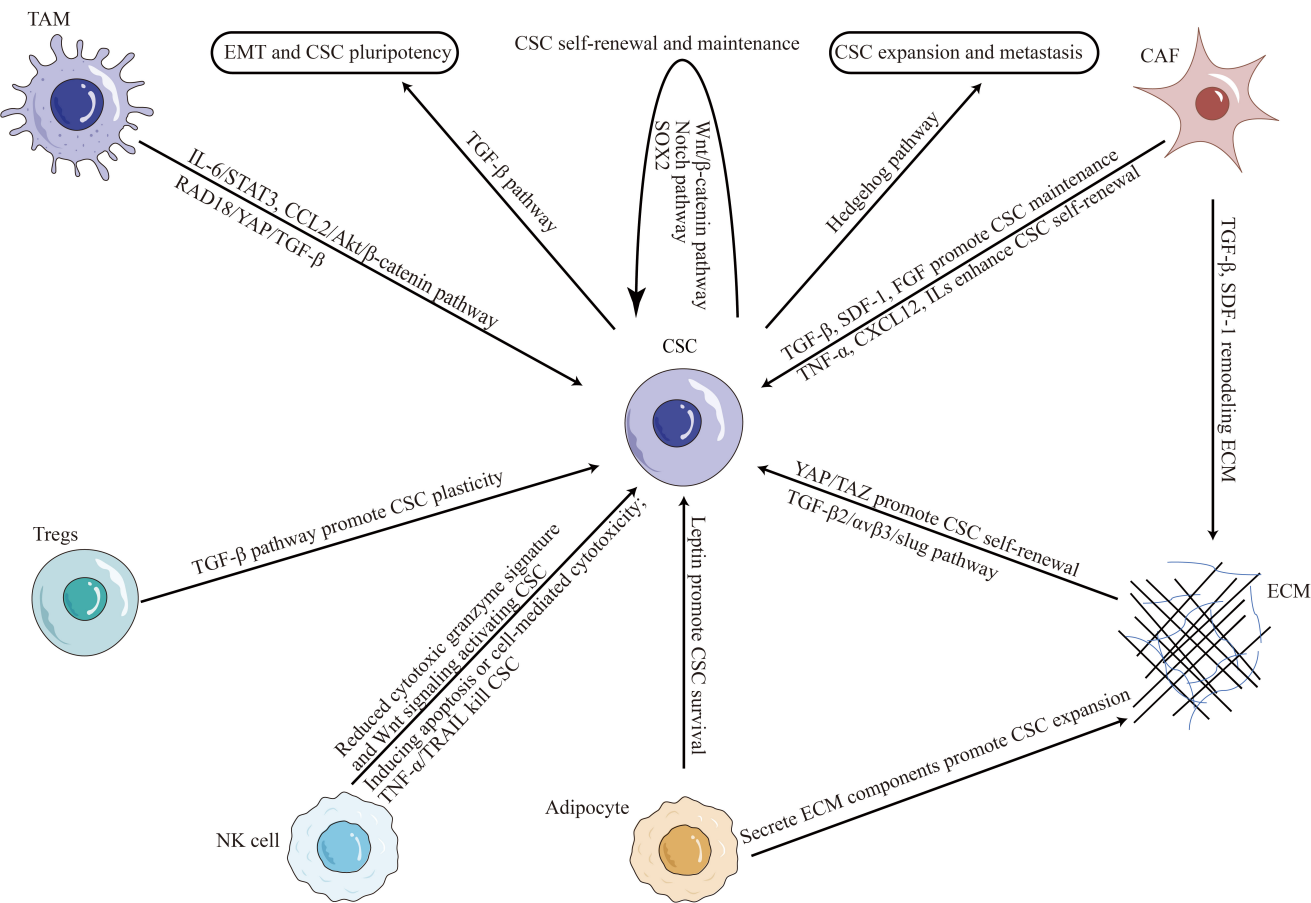
CSC plasticity in TNBC microenvironment. TAM: tumor-associated macrophage; IL-6: interleukin-6; SDF-1: stromal cell-derived factor-1; STAT3: signal transducer and activator of transcription 3; CCL2: C-C motif chemokine ligand 2; RAD18: E3 ubiquitin-protein ligase Rad18; YAP: yes-associated protein; FGF: fibroblast growth factor; TNF-α: tumor necrosis factor (TNF)-alpha; CXCL12: C-X-C motif chemokine ligand 12; TAZ: transcriptional coactivator with PDZ-binding motif; ECM: extracellular matrix; NK: natural killer; TRAIL: TNF-related apoptosis-inducing ligand; Tregs: regulatory T cells

In addition to their intrinsic properties, TNBC CSCs are regulated by their microenvironment. The TME plays a critical role in modulating the survival and growth of CSCs in TNBC (Figure 1).

The TME is a complex system composed of ECM, immune cells, stromal cells, and soluble factors, which all interact to promote tumor growth and progression. It has been shown to drive the generation and maintenance of CSCs by providing survival and self-renewal signals [32, 43]. ECM proteins such as collagen and laminin have been shown to promote CSC expansion and drug resistance [44, 45]. Stromal cells, such as CAFs and endothelial cells, promote TNBC CSC self-renewal and maintenance by secreting cytokines and growth factors [46–48]. Cytokines within the TME, such as the levels of interferon-β and oncostatin-M, modulate CSCs plasticity in TNBC [49]. The reciprocal interaction between CSCs and infiltrating immune cells, such as TAMs, myeloid-derived suppressor cells (MDSCs), dendritic cells (DCs) and others, contribute to TNBC CSC properties by suppressing antitumor immunity and promoting tumor growth and invasion [50].

### Immune cells and CSCs

In recent years, there has been growing interest in the crosstalk between immune cells and CSCs in TNBC. Immune cells, such as TAMs, Tregs, and NK cells, play a critical role in the immune microenvironment of TNBC [51]. Studies have shown that TAMs and Tregs can promote the expansion of CSCs in TNBC by providing survival and self-renewal signals [33, 52]. In contrast, NK cells have been shown to suppress CSCs in TNBC by inducing apoptosis or activating cell-mediated cytotoxicity [53]. However, CSCs also can recruit NK cells into peritumoral microenvironment, but suppress NK cell cytotoxicity through regulating ligands for NK cell activation [54].

Several mechanisms have been proposed to explain the regulation of CSCs by immune cells in TNBC. TAMs, for example, have been shown to secrete cytokines such as IL-6, which activates STAT3 signaling pathways in CSCs, promoting their self-renewal and maintenance [55, 56]. Conversely, Tregs can secrete TGF-β, which promotes CSC plasticity and drug resistance [27]. NK cells, meanwhile, can directly target and kill CSCs via the activation of TRAIL receptors [57]. Tumor-infiltrating NK cells expressed a reduced cytotoxic granzyme signature and were responsible for activating CSCs through Wnt signaling [58].

### Stromal cells and CSCs

Stromal cells, including CAFs, adipocytes, endothelial cells, mesenchymal stem cells, and others, play a critical role in the TME of TNBC. Accumulating evidence suggests that stromal cells can modulate the properties of CSCs in TNBC and contribute to disease progression [59].

CAFs are the most abundant stromal cells in TNBC, and they regulate tumor development through paracrine signaling and ECM remodeling [60]. CAFs have been shown to promote the expansion and maintenance of CSCs in TNBC by secreting growth factors such as TGF-β, SDF-1, FGF, and others [61, 62]. CAFs can secrete a variety of cytokines and chemokines, including CXCL12, TNF-α, and several ILs, and others, promote the reprogramming of cancer cells into CSCs and enhance the self-renewal potential of tumor stem cells [63, 64].

Adipocytes, also present in the stromal compartment of TNBC, have been shown to promote the survival and self-renewal of CSCs through the secretion of leptin and other adipokines [65]**.** Targeting the interaction between stromal cells and CSCs in TNBC has emerged as a potential therapeutic strategy. Several preclinical studies have shown that inhibition of CAFs or adipocytes can reduce CSC populations and improve therapeutic outcomes [5, 62, 65].

### ECM and CSCs

ECM is a non-cellular three-dimensional macromolecular network of proteins and glycosaminoglycans that surrounds cells and provides structural support to tissues [66]. In TNBC, ECM components, such as collagens, fibronectin, and hyaluronan, are dynamically altered and contribute to disease progression [67].

Recent studies have highlighted the importance of ECM-CSC interactions in promoting tumor growth and metastasis in TNBC [68]. ECM stiffness, for example, has been shown to promote CSC self-renewal and tumor-initiating ability by activating YAP/TAZ signaling [69, 70]. Moreover, ECM remodeling by CAFs has been shown to enhance the survival and proliferation of CSCs in TNBC through the secretion of growth factors such as TGF-β and SDF-1 [71]. Adipocytes, another component of the stromal compartment of TNBC, have also been shown to secrete ECM components that promote the expansion of CSCs [72].

ECM-integrin signaling has also been implicated in regulating CSC properties in TNBC [73, 74]. Integrins are transmembrane receptors that mediate cell-ECM interactions and activate downstream signaling pathways. In TNBC, integrin αvβ3 is necessary and sufficient for the CSC phenotype through TGF-β2/αvβ3/slug pathway [75], and the integrin subunits β1 and β3 are used as CSC markers [76]. Furthermore, αvβ3 also interacts with the epidermal growth factor receptor (EGFR) to regulate integrin binding to extracellular ligands required for vasculogenesis and tumor growth [77]. Integrin α6Bβ1 upregulates the expression of a critical stem cell factor, B-cell-specific Moloney murine leukemia virus integration site 1 (BMI-1), by activating focal adhesion kinase (FAK) signaling to contribute to CSC function in TNBC [78].

## Therapies targeting CSCs plasticity in TNBC

Targeting CSCs has emerged as a promising therapeutic approach for TNBC. However, the plasticity of CSCs poses a significant challenge to developing effective treatments. Recent studies have highlighted the dynamic nature of the CSC phenotype in TNBC [79, 80]. CSCs can undergo phenotypic changes in response to microenvironmental cues, enabling them to adapt to changing conditions and evade therapeutic interventions [81, 82]. This plasticity has been linked to the activation of signaling pathways such as Wnt, Notch, and TGF-β, which regulate CSC self-renewal, differentiation, and survival [83–85]. Recent advancements in CSCs-targeted therapies include: chimeric antigen receptor (CAR) therapy, immune cell-based vaccines, oncolytic virotherapy, immune checkpoint inhibitor, antibody-based targeting specific cell surface molecules, energy metabolic enzyme inhibitors, signal pathway inhibitors, nanoparticle-based approaches, combination therapies, and other [86–88]. However, the above therapies may cause side effects like off-target activity, non-specific effect, cytokine release syndrome, hyperbilirubinemia, and other [86].

### Inhibiting signaling pathways that regulate CSC plasticity

The plasticity of CSCs has been linked to the activation of multiple signaling pathways such as Wnt, Notch, Hedgehog, and TGF-β, which regulate CSC self-renewal, differentiation, and survival [89]. Therefore, one approach is to inhibit the signaling pathways that regulate CSC plasticity in TNBC [8].

The Wnt/β-catenin signaling plays an essential role in cell proliferation, differentiation, growth, and survival. In TNBC, aberrant activation of the Wnt/β-catenin pathway has been reported to promote the expansion of CSCs and contribute to therapy resistance [90]. Considerable numbers of molecular drugs that target the Wnt/β-catenin pathway have been studied in the preclinical stage or entered the phase I or II trial stage, including monoclonal antibodies (mAbs), porcupine inhibitors, tankyrase inhibitors, T cell factor (TCF)/β-catenin inhibitors, cAMP-response-element-binding-protein-binding protein (CBP)/β-catenin inhibitors, and B-cell lymphoma 9 (BCL9)/β-catenin inhibitors [91]. For example, PRI-724, a small molecule inhibitor that targets the interaction between β-catenin and CBP, has been shown to suppress CSC self-renewal and inhibit tumor growth in TNBC xenograft models [92, 93]. Similarly, CWP232228, another small molecule inhibitor that blocks the binding of β-catenin to TCF/lymphoid enhancer-binding factor (LEF) transcription factors, inhibits the growth of the CSC population and attenuates insulin-like growth factor-I (IGF-I)-mediated breast CSC (BCSC) functions [94].

Notch signaling is activated by binding Notch ligands to receptors on neighboring cells, leading to the proteolytic cleavage of Notch receptors and the release of the Notch intracellular domain (NICD) [95]. NICD translocates to the nucleus, interacting with transcription factors to activate the expression of target genes that regulate cell fate decisions such as self-renewal and differentiation [96]. To date, several clinical studies involved targeting of Notch pathway with either gamma-secretase inhibitors (GSIs) or mAbs against Notch receptors [97], which represent the major therapeutic targets of the Notch signaling pathway. For example, GSIs block the cleavage of Notch receptors and prevent the release of the NICD, leading to inhibition of the Notch pathway. GSIs have been shown to reduce CSC populations and sensitize TNBC cells to chemotherapy [98]. Similarly, mAbs targeting Notch ligands have been shown to inhibit CSC self-renewal and reduce tumor growth in TNBC xenograft models [99].

The Hedgehog signaling pathway is activated by binding Hedgehog ligands to transmembrane receptors, leading to downstream activation of the transcription factors glioma-associated oncogene homolog 1 (Gli1), Gli2, and Gli3 [100]. Hedgehog signaling is activated in human mammary stem cells, and several Hedgehog inhibitors have been developed and tested in preclinical models [101]. For example, vismodegib, a small-molecule inhibitor (SMI) that targets the Hedgehog pathway by binding to smoothened, has reduced the CSC population and sensitized TNBC cells to chemotherapy [48, 102]. The non-canonical Hedgehog inhibitor Gli-antagonist 61 (GANT61) decreased the TNBC CSC population and enhanced paclitaxel efficacy in TNBC cells [103]. A trans-Pt (II) hedgehog pathway inhibitor complex reduced the number of CSC mammospheres formed [104].

The PI3K/Akt/mechanistic target of rapamycin (mTOR) pathway is one of the essential and active pathways involved in the chemoresistance and survival of TNBC. Inhibitors targeting different components of the pathway have been developed and tested in preclinical models, such as inhibitors of PI3K, Akt, and mTOR [105]. Gedatolisib (PKI-587) is a highly potent dual inhibitor of PI3Kα, PI3Kγ, and mTOR. A phase 1B open-label study of gedatolisib showed an acceptable tolerability profile, with clinical activity at the recommended phase 2 dose in patients with TNBC [106]. PI3K/Akt/mTOR pathway-directed therapy is warranted in treating mesenchymal TNBC, which is enriched in EMT and CSC features. Many trials on combination treatment with PI3K/Akt/mTOR inhibitors and chemotherapy have also been conducted on TNBC, showing promising results [107].

TGF-β has increased stem-like properties in human breast cancer cells [108], as indicated by mammosphere formation and CSC markers. Meanwhile, subpopulations with CSC features (CD44^+^) within breast tumors overexpress TGF-β1 and the TGF-β type I receptor (TGF-βR1) [42]. Inhibition of TGF-β signaling has also been shown to reduce CSC populations and improve therapeutic outcomes [109]. CLK4 inhibition repressed the invasive and CSC properties induced by the TGF-β signaling in TNBC [110]. Several anti-TGF-β approaches, such as TGF-β antibodies, antisense oligonucleotides (ASOs), and SMIs of TβRI kinase, have shown great promise in preclinical studies [111].

### Targeting MTE to regulate CSC plasticity

Targeting the TME has emerged as a potential therapeutic strategy for reducing plasticity and improving therapeutic outcomes in TNBC [112]. Approaches have been developed to target various microenvironment components in preclinical models.

The ECM has been shown to modulate CSC properties by regulating their interaction with stromal cells and growth factors [67]. Targeting the ECM-CSC axis has emerged as a potential therapeutic strategy for TNBC. Integrins serve as ECM-cytoskeletal linkers and transduce biochemical and mechanical signals between cells and their environment [113]. Integrins regulate CSC and tumor stemness in tumors including TNBC, and targeting integrins by mAbs, small molecule inhibitors (including short peptides and natural products) demonstrates efficacy to some extent by reducing CSC populations and improve therapeutic outcomes in TNBC [74]. Fibroblast activation into CAFs is another stroma modification in TME. Therapies targeting CAF activation, CAF reprogramming, CAF-secreted ECM or CAF/tumor cell crosstalk by vaccine, inhibitor (such as IPI-926, defactinib4, C-X-C chemokine receptor type 4 (CXCR4) inhibitor, PEGPH20, matrix metalloproteinase-9 (MMP-9) inhibitor, SOM230, and other) alone or combined with chemotherapy have been evaluated in preclinical studies or clinical trials in the regulation of TNBC initiation and progression [114].

STAT proteins maintain stemness properties and act as mediators of crosstalk between CSCs and infiltrating immune cell populations in the TME. Disulfiram suppresses stem-like properties in TNBC by targeting the STAT3 signaling pathway [115]. Numerous STAT and Janus kinase (JAK) inhibitors, antibodies, and cytokines regulating the JAK-STAT pathway have been developed, and several are currently being assessed in clinical trials [116]. TAMs enhance the stem cell-like properties of cancer cells by upregulating protein S100 calcium-binding protein A9 [117]. TAMs promote EMT and enhance CSC properties in TNBC via activation of CCL2/Akt/β-catenin signaling [118]. RAD18-YAP-TGF-β between TNBC and macrophages regulates cancer stemness and progression [119]. Inhibition of TAM recruitment or activation has been shown to reduce CSC plasticity and improve therapeutic efficacy [120]. CCL2 plays a vital role in the generation of TAMs and is related to the growth of TNBC tumors; inhibiting CCL2 can block tumor stem cell renewal and M2 recruitment, thereby inhibiting the progression of TNBC [121]. Therapies of targeting TAMs in TME, including depletion of M2-like TAMs, blocking TAMs recruitment [colony-stimulating factor-1 (CSF-1)/CSF-1 receptor (CSF-1R) axis, CCL2/C-C motif chemokine receptor 2 (CCR2) axis, neddylation modification], and TAMs reprogramming [toll-like receptors (TLRs) agonists, photodynamic therapy, and other] alone or in combination with chemotherapy or immunotherapy [122].

Immunotherapy has also been explored as a potential targeted therapy for CSCs in TNBC. Immune-based therapies such as CAR-T cells targeting CSCs in TNBC are also being developed, and trophoblast cell surface antigen 2 (TROP2), disialoganglioside GD2 (GD2), receptor tyrosine kinase-like orphan receptor 1 (ROR1), mucin 1 (MUC1), and EpCAM promise targets [123]. Immune checkpoint inhibitors alone show no efficiency in stem cell-like TNBC, but they can activate the immune system to restrain CSCs in combination with messenger RNA (mRNA) ligand-dependent corepressor (LCOR) therapy [124]. Besides mAbs, vaccines, and small molecules such as ruxolitinib or repair have also been used to target the CSC/immune-microenvironment interaction in clinical trials [125].

### Other promising CSC-targeted therapies

In addition to targeting signaling pathways and the microenvironment, metabolic plasticity has emerged as a potential therapeutic target for CSCs. CSCs exhibit metabolic flexibility, which enables them to adapt to changing nutrient availability and microenvironmental stresses [126]. CSCs have a unique metabolic phenotype that switches between glycolysis and oxidative phosphorylation (OXPHS) to adapt to nutrient deficiency and therapeutic stress [127]. The cholesterol biosynthesis pathway has recently been identified as crucial in the proliferation, survival, and differentiation of CSCs in TNBC [16]. Furthermore, accumulating evidence has linked the metabolic plasticity of CSCs to their increased resistance to therapy [128]. Therefore, targeting metabolic pathways has been shown to reduce the CSC population and improve treatment outcomes in TNBC [129].

**Figure 2:**
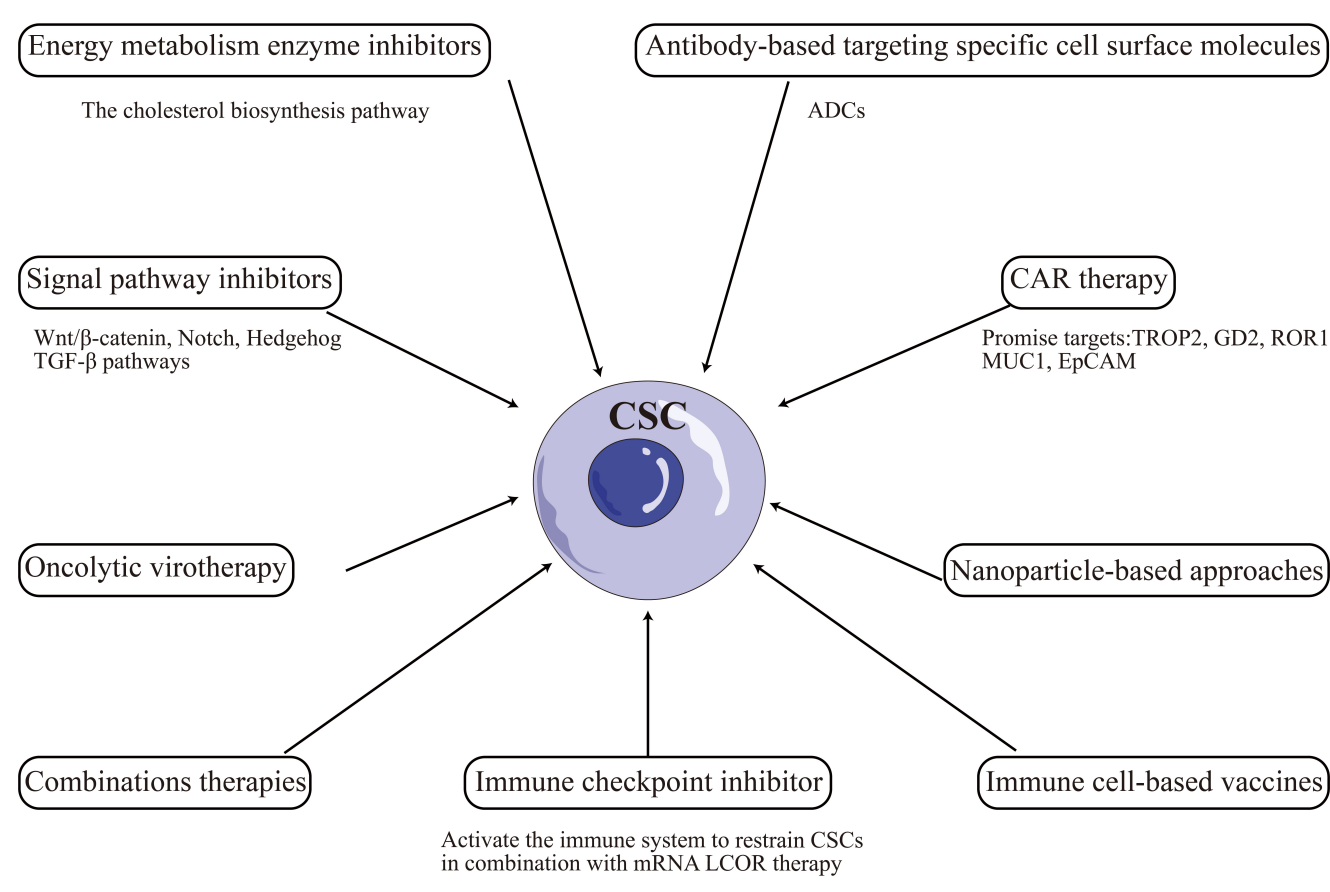
CSCs-targeted therapies

Other promising CSC-targeted therapies are being developed, including small molecule inhibitors, mAbs, and other [130]. The TKI inhibitor dasatinib has shown efficacy in targeting CSCs in TNBC by blocking CSC enrichment and Src activation, inducing EMT, and enhancing sensitivity to paclitaxel [131]. Similarly, mAbs targeting the CSC markers CD47 or ROR1 have shown efficacy against TNBC in preclinical models [132–134]. Antibody-drug conjugates (ADCs) are a novel strategy for targeting CSCs in TNBC, which consists of an antibody that targets a specific antigen on the surface of cancer cells, linked to a cytotoxic drug that kills the targeted cells. ADCs have been designed to target CSC-specific markers such as CD44 and EpCAM [135]. Overall, the CSCs-targeted therapies illustrated in this paper were summarized and visualized in Figure 2.

## Prospects and challenges of targeting CSC plasticity in TNBC treatment

CSCs have been identified as critical contributors to TNBC pathogenesis due to their self-renewal capacity, ability to initiate tumor growth, and resistance to therapy. The prospects and challenges of treating TNBC by targeting CSC plasticity will be discussed in this section.

One potential strategy for targeting CSC plasticity is inhibiting EMT, a process by which epithelial cells acquire mesenchymal properties and become more invasive and resistant to therapy. Several signaling pathways, including TGF-β, Wnt, Notch, and Hedgehog, have been implicated in EMT regulation and targeted for TNBC therapy [136–138]. For instance, it has been shown that the inhibition of TGF-β signaling can prevent EMT and suppress the growth and metastasis of TNBC [136]. Additionally, the inhibition of the Wnt pathway has been reported to decrease the self-renewal capacity of CSCs and enhance their sensitivity to chemotherapy [138]. However, the clinical translation of these findings remains a major challenge, and further studies are needed to determine the most effective and safe ways to target EMT-mediated CSC plasticity in TNBC.

Another potential approach for targeting CSC plasticity is through combinatorial therapies that target multiple cell states of CSCs. For example, a recent study suggested that simultaneous targeting of both CSCs and non-CSCs with chemotherapy and immunotherapy may improve treatment outcomes in TNBC [21]**.** Additionally, targeting the crosstalk between CSCs and the TME, such as CAFs, immune cells, and ECM, may provide a novel approach for TNBC therapy [139]. Although some progress has been achieved during practice, developing these combination strategies still requires careful consideration of their potential toxicities and optimization of treatment regimens.

Furthermore, recent studies have also highlighted the critical role of metabolic reprogramming in CSC plasticity [140, 141]. Metabolic pathways, such as glucose metabolism and mitochondrial respiration, have been shown to play a crucial role in CSC maintenance and response to therapy [142]. Thus, targeting metabolic pathways may represent a promising approach for improving TNBC treatment outcomes.

Despite the promising results obtained from preclinical studies, several challenges must be addressed before targeting CSC plasticity can be effectively translated into clinical practice. Firstly, the heterogeneity and plasticity of CSCs make their identification and targeting difficult. Secondly, the potential toxicity of CSC-targeted therapies to normal stem cells needs to be carefully evaluated. Thirdly, side effects like the off-target activity, cytokine release syndrome should be considered when adopting the immune-based therapy. Finally, optimal combinations of CSC-targeted therapy with other treatments, such as chemotherapy and radiotherapy, need to be determined.

## Conclusion

In conclusion, targeting CSC’s plasticity represents a promising approach to treating TNBC. Strategies for engineering immune effector cell differentiation, inhibiting signaling pathways, disrupting the microenvironment, and targeting metabolic pathways may reduce the CSC population and improve therapeutic efficacy. Future research should focus on identifying novel targets and developing novel therapeutic strategies to target CSC’s plasticity in TNBC treatment.

## Abbreviations

ADCs: antibody-drug conjugates
Akt: protein kinase B
CAFs: cancer-associated fibroblasts
CAR: Chimeric antigen receptor
CCL2: C-C motif chemokine ligand 2
CSCs: cancer stem cells
CXCL12: C-X-C motif chemokine ligand 12
ECM: extracellular matrix
EMT: epithelial-mesenchymal transition
EpCAM: epithelial cell adhesion molecule
FGF: fibroblast growth factor
GSIs: gamma-secretase inhibitors
IL-6: interleukin-6
mAbs: monoclonal antibodies
mTOR: mechanistic target of rapamycin
NICD: Notch intracellular domain
NK: natural killer
PI3K: phosphatidylinositol 3-kinase
RAD18: E3 ubiquitin-protein ligase Rad18
ROR1: receptor tyrosine kinase-like orphan receptor 1
SDF-1: stromal cell-derived factor-1
STAT3: signal transducer and activator of transcription 3
TAMs: tumor-associated macrophages
TAZ: transcriptional coactivator with PDZ-binding motif
TGF-β: transforming growth factor-β
TME: tumor microenvironment
TNBC: triple-negative breast cancer
TNF: tumor necrosis factor
TRAIL: tumor necrosis factor-related apoptosis-inducing ligand
Tregs: regulatory T cells
YAP: yes-associated protein

## Declarations

### Copyright

© The Author(s) 2023.
